# Supplementary material for: Software-aided workflow for predicting protease-specific cleavage sites using physicochemical properties of the natural and unnatural amino acids in peptide-based drug discovery
Source: PLoS One. 2019 Jan 8;14(1):e0199270. doi: 10.1371/journal.pone.0199270 (PMC6324806; doi:10.1371/journal.pone.0199270)
Supplement: S1 Table — (PDF) [file pone.0199270.s001.pdf]

Supporting Table 1. Summary on the extracted information from MEROPS database

Protease family	Protease	Substrates	Cleavage events	SoC2 P1-P1’	SoC8 P4-P4’	Substrates in external dataset
Serine proteases	Granzyme B (rodent-type)	31	32	11	15	7
	Trypsin 1	327	1104	43	847	31
	Granzyme M	72	164	47	70	7
	Granzyme A	66	135	18	40	14
	Granzyme B	65	121	46	73	17
	Thrombin	53	59	14	39	12
Matrix metallopeptidases	Matrix Metallopeptidase-2	1359	1528	199	1057	53
	Matrix Metallopeptidase-3	11	29	21	23	4
	Matrix Metallopeptidase-8	11	25	16	16	3
	Matrix Metallopeptidase-9	20	185	58	74	2
Aspartic proteases	Cathepsin D	16	53	37	38	5
	Cathepsin E	8	20	11	14	3
Cysteine Aspartate proteases	Caspase-1	10	10	6	8	2
	Caspase-2	216	221	17	136	44
	Caspase-3	52	72	13	42	10
	Caspase-6	870	885	25	782	10
	Caspase-7	23	29	6	13	6
Cysteine proteases	Cathepsin L	895	964	209	587	186
