# Supplementary material for: Software-aided workflow for predicting protease-specific cleavage sites using physicochemical properties of the natural and unnatural amino acids in peptide-based drug discovery
Source: PLoS One. 2019 Jan 8;14(1):e0199270. doi: 10.1371/journal.pone.0199270 (PMC6324806; doi:10.1371/journal.pone.0199270)
Supplement: S2 Table — (PDF) [file pone.0199270.s002.pdf]

Supporting Table 2. Summary on the model's number for each protease for cleavage window P1-P1' and P4-P4'

Protease	Models number for SoC2	Models number for SoC8
**Granzyme B (rodent-type)**	32	126
**Trypsin 1**	13	38
**Granzyme M**	8	79
**Granzyme A**	21	94
**Granzyme B**	8	102
**Thrombin**	24	21
**Matrix Metallopeptidase-2**	2	14
**Matrix Metallopeptidase-3**	11	26
**Matrix Metallopeptidase-8**	15	38
**Matrix Metallopeptidase-9**	4	16
**Cathepsin D**	7	28
**Cathepsin E**	7	5
**Caspase-1**	57	148
**Caspase-2**	27	25
**Caspase-3**	31	54
**Caspase-6**	25	23
**Caspase-7**	33	27
**Cathepsin L**	2	14
