# Supplementary material for: Software-aided workflow for predicting protease-specific cleavage sites using physicochemical properties of the natural and unnatural amino acids in peptide-based drug discovery
Source: PLoS One. 2019 Jan 8;14(1):e0199270. doi: 10.1371/journal.pone.0199270 (PMC6324806; doi:10.1371/journal.pone.0199270)
Supplement: S3 Table — (PDF) [file pone.0199270.s003.pdf]

Supporting Table 3. Summary on count of individual SoC2 and SoC8 calculated by frequency analysis

Protease	Datasets for window P1-P1'				Datasets for window P4-P4'			
	Train dataset size		External dataset size		Train dataset size		External dataset size	
	Negative SoCs	Positive SoCs	Negative SoCs	Positive SoCs	Negative SoCs	Positive SoCs	Negative SoCs	Positive SoCs
**Granzyme B (rodent-type)**	371	11	270	4	1923	15	920	7
**Trypsin 1**	633	43	434	33	34217	847	4031	113
**Granzyme M**	458	47	313	11	5676	70	956	18
**Granzyme A**	420	18	342	10	3856	40	1910	27
**Granzyme B**	471	46	396	15	7558	73	2323	24
**Thrombin**	363	14	146	8	915	39	154	11
**Matrix Metallopeptidase-2**	718	199	424	48	17017	1057	1059	54
**Matrix Metallopeptidase-3**	278	21	242	6	651	23	362	6
**Matrix Metallopeptidase-8**	288	16	85	5	636	16	79	2
**Matrix Metallopeptidase-9**	376	58	211	19	1365	74	400	21
**Cathepsin D**	348	37	213	11	1129	38	346	11
**Cathepsin E**	102	11	192	6	94	14	264	6
**Caspase-1**	356	6	188	2	1200	8	312	2
**Caspase-2**	491	17	320	7	3610	136	961	36
**Caspase-3**	435	13	356	7	2361	42	1421	14
**Caspase-6**	668	25	259	6	19229	782	574	11
**Caspase-7**	212	6	234	2	382	13	417	5
**Cathepsin L**	765	209	576	118	9479	587	2726	174
