# Supplementary material for: Software-aided workflow for predicting protease-specific cleavage sites using physicochemical properties of the natural and unnatural amino acids in peptide-based drug discovery
Source: PLoS One. 2019 Jan 8;14(1):e0199270. doi: 10.1371/journal.pone.0199270 (PMC6324806; doi:10.1371/journal.pone.0199270)
Supplement: S4 Table — (PDF) [file pone.0199270.s004.pdf]

S4 Table. The predictive performance evaluation for Logistic Regression and Support Vector Machine Classifiers based on results of the 5-fold Cross validation for caspases and cathepsin L.

		Cleavage site size P1-P1'						Cleavage site size P4-P4'					
		caspase1	caspase2	caspase3	caspase6	caspase7	cathepsinL	caspase1	caspase2	caspase3	caspase6	caspase7	cathepsinL
Accuracy	LR	0.77	0.89	0.90	0.94	0.77	0.83	0.73	0.93	0.88	0.96	0.87	0.84
	SVC	0.89	0.90	0.90	0.93	0.83	0.81	0.67	0.86	0.79	0.90	0.68	0.77
AUC ROC	LR	0.77	0.87	0.90	0.92	0.77	0.81	0.73	0.93	0.88	0.96	0.87	0.84
	SVC	0.89	0.89	0.91	0.92	0.83	0.76	0.67	0.86	0.79	0.90	0.68	0.77
MCC	LR	0.58	0.78	0.82	0.86	0.58	0.62	0.50	0.87	0.77	0.92	0.76	0.69
	SVC	0.80	0.81	0.82	0.86	0.69	0.54	0.37	0.74	0.63	0.80	0.36	0.54
AUC PRC	LR	0.73	0.87	0.89	0.93	0.76	0.84	0.72	0.90	0.83	0.94	0.84	0.79
	SVC	0.87	0.88	0.90	0.94	0.83	0.83	0.67	0.85	0.78	0.88	0.68	0.72
Sensitivity	LR	0.96	0.95	0.97	0.98	0.92	0.88	0.80	0.96	0.93	0.97	0.95	0.82
	SVC	0.92	0.90	0.91	0.94	0.83	0.87	0.55	0.76	0.62	0.84	0.37	0.68
Specificity	LR	0.59	0.79	0.82	0.86	0.62	0.73	0.66	0.90	0.83	0.95	0.79	0.87
	SVC	0.85	0.87	0.90	0.91	0.83	0.65	0.78	0.96	0.97	0.96	0.98	0.86
