# Supplementary material for: Software-aided workflow for predicting protease-specific cleavage sites using physicochemical properties of the natural and unnatural amino acids in peptide-based drug discovery
Source: PLoS One. 2019 Jan 8;14(1):e0199270. doi: 10.1371/journal.pone.0199270 (PMC6324806; doi:10.1371/journal.pone.0199270)
Supplement: S5 Table — (PDF) [file pone.0199270.s005.pdf]

S5 Table. The predictive performance evaluation for Logistic Regression and Support Vector Machine Classifiers based on results of the external validation for caspases and cathepsin L.

Learning algorithm	Local window size	Performance metrics	caspase1	caspase2	caspase3	caspase6	caspase7	cathepsinL
**LR**	**P1/P1'**	**Accuracy**	0.68	0.83	0.80	0.82	0.70	0.33
		**AUC PRC**	0.14	0.49	0.23	0.40	0.25	0.14
		**AUC ROC**	0.03	0.31	0.08	0.22	0.10	0.09
		**MCC**	0.84	0.91	0.87	0.91	0.83	0.62
		**Sensitivity**	1.00	1.00	0.94	1.00	0.97	0.95
		**Specificity**	0.68	0.82	0.79	0.81	0.69	0.29
	**P4/P4'**	**Accuracy**	0.69	0.76	0.84	0.75	0.64	0.72
		**AUC PRC**	0.10	0.53	0.22	0.46	0.22	0.41
		**AUC ROC**	0.02	0.43	0.09	0.36	0.10	0.33
		**MCC**	0.77	0.77	0.84	0.74	0.68	0.73
		**Sensitivity**	0.86	0.79	0.85	0.74	0.73	0.73
		**Specificity**	0.69	0.75	0.83	0.75	0.64	0.73
**SVC**	**P1/P1'**	**Accuracy**	0.83	0.85	0.97	0.83	0.85	0.33
		**AUC PRC**	0.21	0.50	0.85	0.40	0.38	0.13
		**AUC ROC**	0.05	0.33	0.27	0.22	0.20	0.09
		**MCC**	0.92	0.92	0.10	0.91	0.90	0.62
		**Sensitivity**	1.00	1.00	0.91	1.00	0.96	0.94
		**Specificity**	0.83	0.84	0.84	0.82	0.84	0.29
	**P4/P4'**	**Accuracy**	0.74	0.77	0.88	0.76	0.72	0.73
		**AUC PRC**	0.12	0.55	0.26	0.50	0.29	0.37
		**AUC ROC**	0.02	0.46	0.11	0.39	0.17	0.32
		**MCC**	0.82	0.77	0.86	0.74	0.71	0.69
		**Sensitivity**	0.90	0.78	0.83	0.72	0.70	0.63
		**Specificity**	0.74	0.77	0.88	0.76	0.72	0.74
