# Supplementary material for: Software-aided workflow for predicting protease-specific cleavage sites using physicochemical properties of the natural and unnatural amino acids in peptide-based drug discovery
Source: PLoS One. 2019 Jan 8;14(1):e0199270. doi: 10.1371/journal.pone.0199270 (PMC6324806; doi:10.1371/journal.pone.0199270)
Supplement: S6 Table — (PDF) [file pone.0199270.s006.pdf]

S6 Table. The predictive performance evaluation for Logistic Regression and SVC Classifiers based on results of the external validation for all proteases P1/P1'.						
**Learning algorithm**	**LR**					
**Local window size**	**P1/P1'**					
**Performance metrics**	**Accuracy**	**AUC PRC**	**AUC ROC**	**MCC**	**Sensitivity**	**Specificity**
**caspase1**	0.68	0.14	0.03	0.84	1.00	0.68
**caspase2**	0.83	0.49	0.31	0.91	1.00	0.82
**caspase3**	0.80	0.23	0.08	0.87	0.94	0.79
**caspase6**	0.82	0.40	0.22	0.91	1.00	0.81
**caspase7**	0.70	0.25	0.10	0.83	0.97	0.69
**cathepsinD**	0.62	0.15	0.07	0.70	0.80	0.61
**cathepsinE**	0.51	0.11	0.05	0.68	0.85	0.50
**cathepsinL**	0.33	0.14	0.09	0.62	0.95	0.29
**granzymeA**	0.61	0.20	0.06	0.75	0.98	0.63
**granzymeB**	0.47	0.09	0.03	0.71	0.96	0.46
**granzymeBrt**	0.56	0.11	0.02	0.78	1.00	0.55
**granzymeM**	0.53	0.13	0.05	0.72	0.92	0.52
**MMP2**	0.40	0.16	0.09	0.66	0.96	0.36
**MMP3**	0.53	0.06	0.06	0.58	0.64	0.53
**MMP8**	0.54	0.25	0.22	0.71	0.94	0.48
**MMP9**	0.55	0.12	0.11	0.64	0.74	0.53
**thrombin**	0.81	0.45	0.27	0.89	0.99	0.80
**trypsin1**	0.87	0.42	0.22	0.93	1.00	0.87
**Learning algorithm**	**SVC**					
**Local window size**	**P1/P1'**					
**Performance metrics**	**Accuracy**	**AUC PRC**	**AUC ROC**	**MCC**	**Sensitivity**	**Specificity**
**caspase1**	0.83	0.21	0.05	0.92	1.00	0.83
**caspase2**	0.85	0.50	0.33	0.92	1.00	0.84
**caspase3**	0.97	0.85	0.27	0.10	0.91	0.84
**caspase6**	0.83	0.40	0.22	0.91	1.00	0.82
**caspase7**	0.85	0.38	0.20	0.90	0.96	0.84
**cathepsinD**	0.53	0.07	0.06	0.60	0.66	0.53
**cathepsinE**	0.74	0.20	0.09	0.78	0.82	0.74
**cathepsinL**	0.33	0.13	0.09	0.62	0.94	0.29
**granzymeA**	0.70	0.19	0.06	0.84	0.97	0.70
**granzymeB**	0.51	0.09	0.03	0.71	0.92	0.50
**granzymeBrt**	0.80	0.20	0.05	0.89	0.99	0.79
**granzymeM**	0.47	0.11	0.04	0.68	0.90	0.46
**MMP2**	0.42	0.17	0.10	0.67	0.97	0.38
**MMP3**	0.61	0.01	0.05	0.57	0.52	0.63
**MMP8**	0.54	0.24	0.26	0.69	0.89	0.50
**MMP9**	0.63	0.17	0.13	0.68	0.74	0.63
**thrombin**	0.89	0.61	0.44	0.94	1.00	0.89
**trypsin1**	0.89	0.45	0.25	0.94	1.00	0.89
