# Supplementary material for: Software-aided workflow for predicting protease-specific cleavage sites using physicochemical properties of the natural and unnatural amino acids in peptide-based drug discovery
Source: PLoS One. 2019 Jan 8;14(1):e0199270. doi: 10.1371/journal.pone.0199270 (PMC6324806; doi:10.1371/journal.pone.0199270)
Supplement: S7 Table — (PDF) [file pone.0199270.s007.pdf]

S7 Table. The predictive performance evaluation for Logistic Regression and SVC Classifiers based on results of the external validation for all proteases for P4/P4'.						
**Learning algorithm**	**LR**					
**Local window size**	**P4/P4'**					
**Performance metrics**	**Accuracy**	**AUC PRC**	**AUC ROC**	**MCC**	**Sensitivity**	**Specificity**
**caspase1**	0.69	0.10	0.02	0.77	0.86	0.69
**caspase2**	0.76	0.53	0.43	0.77	0.79	0.75
**caspase3**	0.84	0.22	0.09	0.84	0.85	0.83
**caspase6**	S6_	0.46	0.36	0.74	0.74	0.75
**caspase7**	0.64	0.22	0.10	0.68	0.73	0.64
**cathepsinD**	0.64	0.08	0.06	0.62	0.60	0.64
**cathepsinE**	0.58	0.09	0.05	0.67	0.76	0.57
**cathepsinL**	0.72	0.41	0.33	0.73	0.73	0.73
**granzymeA**	0.73	0.17	0.05	0.83	0.93	0.73
**granzymeB**	0.69	0.12	0.04	0.77	0.85	0.69
**granzymeBrt**	0.65	0.12	0.02	0.83	1.00	0.65
**granzymeM**	0.66	0.14	0.04	0.76	0.86	0.66
**MMP2**	0.78	0.49	0.38	0.79	0.82	0.77
**MMP3**	0.59	0.10	0.12	0.62	0.66	0.57
**MMP8**	0.41	0.16	0.15	0.49	0.58	0.39
**MMP9**	0.59	0.09	0.11	0.60	0.62	0.59
**thrombin**	0.85	0.69	0.58	0.88	0.92	0.84
**trypsin1**	0.88	0.40	0.21	0.88	0.89	0.88
**Learning algorithm**	**SVC**					
**Local window size**	**P4/P4'**					
**Performance metrics**	**Accuracy**	**AUC PRC**	**AUC ROC**	**MCC**	**Sensitivity**	**Specificity**
**caspase1**	0.74	0.12	0.02	0.82	0.90	0.74
**caspase2**	0.77	0.55	0.46	0.77	0.78	0.77
**caspase3**	0.88	0.26	0.11	0.86	0.83	0.88
**caspase6**	0.76	0.50	0.39	0.74	0.72	0.76
**caspase7**	0.72	0.29	0.17	0.71	0.70	0.72
**cathepsinD**	0.64	0.07	0.06	0.61	0.57	0.64
**cathepsinE**	0.57	0.09	0.05	0.67	0.78	0.56
**cathepsinL**	0.73	0.37	0.32	0.69	0.63	0.74
**granzymeA**	0.77	0.19	0.06	0.85	0.93	0.77
**granzymeB**	0.73	0.13	0.04	0.79	0.84	0.73
**granzymeBrt**	0.71	0.14	0.03	0.85	1.00	0.71
**granzymeM**	0.68	0.15	0.05	0.77	0.85	0.68
**MMP2**	0.80	0.43	0.37	0.73	0.65	0.81
**MMP3**	0.72	0.20	0.19	0.69	0.66	0.72
**MMP8**	0.44	0.19	0.18	0.50	0.57	0.43
**MMP9**	0.59	0.09	0.10	0.60	0.61	0.59
**thrombin**	0.85	0.69	0.59	0.88	0.92	0.84
**trypsin1**	0.89	0.36	0.20	0.79	0.69	0.90
