# Supplementary material for: Software-aided workflow for predicting protease-specific cleavage sites using physicochemical properties of the natural and unnatural amino acids in peptide-based drug discovery
Source: PLoS One. 2019 Jan 8;14(1):e0199270. doi: 10.1371/journal.pone.0199270 (PMC6324806; doi:10.1371/journal.pone.0199270)
Supplement: S8 Table — (PDF) [file pone.0199270.s008.pdf]

S8 Table.The performance validation results for all classifiers on external dataset for P4/P4' models.							
**Performance metrics**	**Learning algorithm**	**Accuracy**	**AUC PRC**	**AUC ROC**	**MCC**	**Sensitivity**	**Specificity**
**Caspase1**	**LR**	0.69	0.10	0.02	0.77	0.86	0.69
	**SVC**	0.74	0.12	0.02	0.82	0.90	0.74
	**RFC**	0.85	0.18	0.05	0.88	0.91	0.85
	**GBC**	0.85	0.21	0.05	0.91	0.97	0.85
**Caspase2**	**LR**	0.76	0.53	0.43	0.77	0.79	0.75
	**SVC**	0.77	0.55	0.46	0.77	0.78	0.77
	**RFC**	0.77	0.58	0.48	0.79	0.81	0.77
	**GBC**	0.77	0.57	0.48	0.79	0.81	0.77
**Caspase3**	**LR**	0.84	0.22	0.09	0.84	0.85	0.83
	**SVC**	0.88	0.26	0.11	0.86	0.83	0.88
	**RFC**	0.94	0.36	0.19	0.89	0.84	0.94
	**GBC**	0.94	0.36	0.19	0.89	0.84	0.94
**Caspase6**	**LR**	0.75	0.46	0.36	0.74	0.74	0.75
	**SVC**	0.76	0.50	0.39	0.74	0.72	0.76
	**RFC**	0.76	0.46	0.32	0.74	0.72	0.76
	**GBC**	0.76	0.47	0.32	0.76	0.76	0.76
**Caspase7**	**LR**	0.64	0.22	0.10	0.68	0.73	0.64
	**SVC**	0.72	0.29	0.17	0.71	0.70	0.72
	**RFC**	0.77	0.38	0.24	0.77	0.77	0.77
	**GBC**	0.73	0.35	0.21	0.76	0.79	0.73
**CathepsinD**	**LR**	0.64	0.08	0.06	0.62	0.60	0.64
	**SVC**	0.64	0.07	0.06	0.61	0.57	0.64
	**RFC**	0.70	0.16	0.08	0.70	0.71	0.70
	**GBC**	0.66	0.14	0.07	0.69	0.73	0.66
**CathepsinE**	**LR**	0.58	0.09	0.05	0.67	0.76	0.57
	**SVC**	0.57	0.09	0.05	0.67	0.78	0.56
	**RFC**	0.71	0.15	0.06	0.74	0.78	0.71
	**GBC**	0.63	0.14	0.06	0.73	0.83	0.62
**CathepsinL**	**LR**	0.72	0.41	0.33	0.73	0.73	0.73
	**SVC**	0.73	0.37	0.32	0.69	0.63	0.74
	**RFC**	0.75	0.45	0.37	0.74	0.73	0.75
	**GBC**	0.75	0.47	0.38	0.76	0.78	0.74
**GranzymeA**	**LR**	0.73	0.17	0.05	0.83	0.93	0.73
	**SVC**	0.77	0.19	0.06	0.85	0.93	0.77
	**RFC**	0.85	0.26	0.09	0.90	0.96	0.84
	**GBC**	0.80	0.22	0.07	0.87	0.95	0.80
**GranzymeB**	**LR**	0.69	0.12	0.04	0.77	0.85	0.69
	**SVC**	0.73	0.13	0.04	0.79	0.84	0.73
	**RFC**	0.80	0.16	0.05	0.83	0.86	0.80
	**GBC**	0.74	0.13	0.04	0.81	0.87	0.74
**GranzymeBrt**	**LR**	0.65	0.12	0.02	0.83	1.00	0.65
	**SVC**	0.71	0.14	0.03	0.85	1.00	0.71
	**RFC**	0.84	0.21	0.06	0.92	1.00	0.84
	**GBC**	0.73	0.15	0.03	0.86	1.00	0.72

S8 Table.The performance validation results for all classifiers on external dataset for P4/P4' models.							
**Performance metrics**	**Learning algorithm**	**Accuracy**	**AUC PRC**	**AUC ROC**	**MCC**	**Sensitivity**	**Specificity**
**GranzymeM**	**LR**	0.66	0.14	0.04	0.76	0.86	0.66
	**SVC**	0.68	0.15	0.05	0.77	0.85	0.68
	**RFC**	0.78	0.19	0.06	0.80	0.82	0.78
	**GBC**	0.73	0.19	0.06	0.81	0.90	0.73
**MMP2**	**LR**	0.78	0.49	0.38	0.79	0.82	0.77
	**SVC**	0.80	0.43	0.37	0.73	0.65	0.81
	**RFC**	0.78	0.47	0.37	0.78	0.78	0.77
	**GBC**	0.77	0.51	0.38	0.80	0.84	0.78
**MMP3**	**LR**	0.59	0.10	0.12	0.62	0.66	0.57
	**SVC**	0.72	0.20	0.19	0.69	0.66	0.72
	**RFC**	0.76	0.17	0.16	0.68	0.57	0.78
	**GBC**	0.69	0.12	0.13	0.64	0.57	0.71
**MMP8**	**LR**	0.41	0.16	0.15	0.49	0.58	0.39
	**SVC**	0.44	0.19	0.18	0.50	0.57	0.43
	**RFC**	0.42	0.12	0.15	0.45	0.49	0.42
	**GBC**	0.38	0.01	0.12	0.39	0.38	0.40
**MMP9**	**LR**	0.59	0.09	0.11	0.60	0.62	0.59
	**SVC**	0.59	0.09	0.10	0.60	0.61	0.59
	**RFC**	0.70	0.16	0.13	0.68	0.66	0.71
	**GBC**	0.64	0.16	0.12	0.69	0.74	0.64
**Thrombin**	**LR**	0.85	0.69	0.58	0.88	0.92	0.84
	**SVC**	0.85	0.69	0.59	0.88	0.92	0.84
	**RFC**	0.88	0.77	0.70	0.89	0.91	0.87
	**GBC**	0.84	0.67	0.58	0.86	0.90	0.83
**Trypsin1**	**LR**	0.88	0.40	0.21	0.88	0.89	0.88
	**SVC**	0.89	0.36	0.20	0.79	0.69	0.90
	**RFC**	0.87	0.41	0.22	0.90	0.92	0.87
	**GBC**	0.87	0.40	0.22	0.90	0.92	0.87
