# Supplementary material for: Software-aided workflow for predicting protease-specific cleavage sites using physicochemical properties of the natural and unnatural amino acids in peptide-based drug discovery
Source: PLoS One. 2019 Jan 8;14(1):e0199270. doi: 10.1371/journal.pone.0199270 (PMC6324806; doi:10.1371/journal.pone.0199270)
Supplement: S9 Table — (PDF) [file pone.0199270.s009.pdf]

Supporting Table 9. The percentage of recovered known cleavage sites at percentage of ranking position reached when best recovered 100% of all known sites of cleavage for all selected proteases. Highest recovered percentage marked in bold.

	LR (%)	SVC (%)	RCF (%)	GBC (%)	Best (%)	Random (%)
**Serine protease**	93	91	**96**	**96**	100.0	13
**Cysteine protease**	94	89	94	**95**	100.0	29
**Aspartic protease**	29	29	**47**	35	100.0	0
**Matrix metalloproteases**	**86**	82	**86**	83	100.0	25
