# Supplementary material for: Software-aided workflow for predicting protease-specific cleavage sites using physicochemical properties of the natural and unnatural amino acids in peptide-based drug discovery
Source: PLoS One. 2019 Jan 8;14(1):e0199270. doi: 10.1371/journal.pone.0199270 (PMC6324806; doi:10.1371/journal.pone.0199270)
Supplement: S10 Table — (PDF) [file pone.0199270.s010.pdf]

Supporting Table 10. The normalized ranking percentage position reached by logistic regression and random forest models when 100% of known SoC was recovered for all selected proteases. Lowest ranking percentage position marked in bold.

	**LR (%)**	**SVC (%)**	**RCF (%)**	**GBC (%)**	**Best (%)**	**Random (%)**
**Serine protease**	95	**73**	86	88	12	100
**Cysteine protease**	95	100	88	**84**	30	100
**Aspartic protease**	98	96	**84**	90	7	87
**Matrix metalloproteases**	89	100	90	**87**	25	100
