# Supplementary figures and images for: Software-aided workflow for predicting protease-specific cleavage sites using physicochemical properties of the natural and unnatural amino acids in peptide-based drug discovery
Source: PLoS One. 2019 Jan 8;14(1):e0199270. doi: 10.1371/journal.pone.0199270 (PMC6324806; doi:10.1371/journal.pone.0199270)

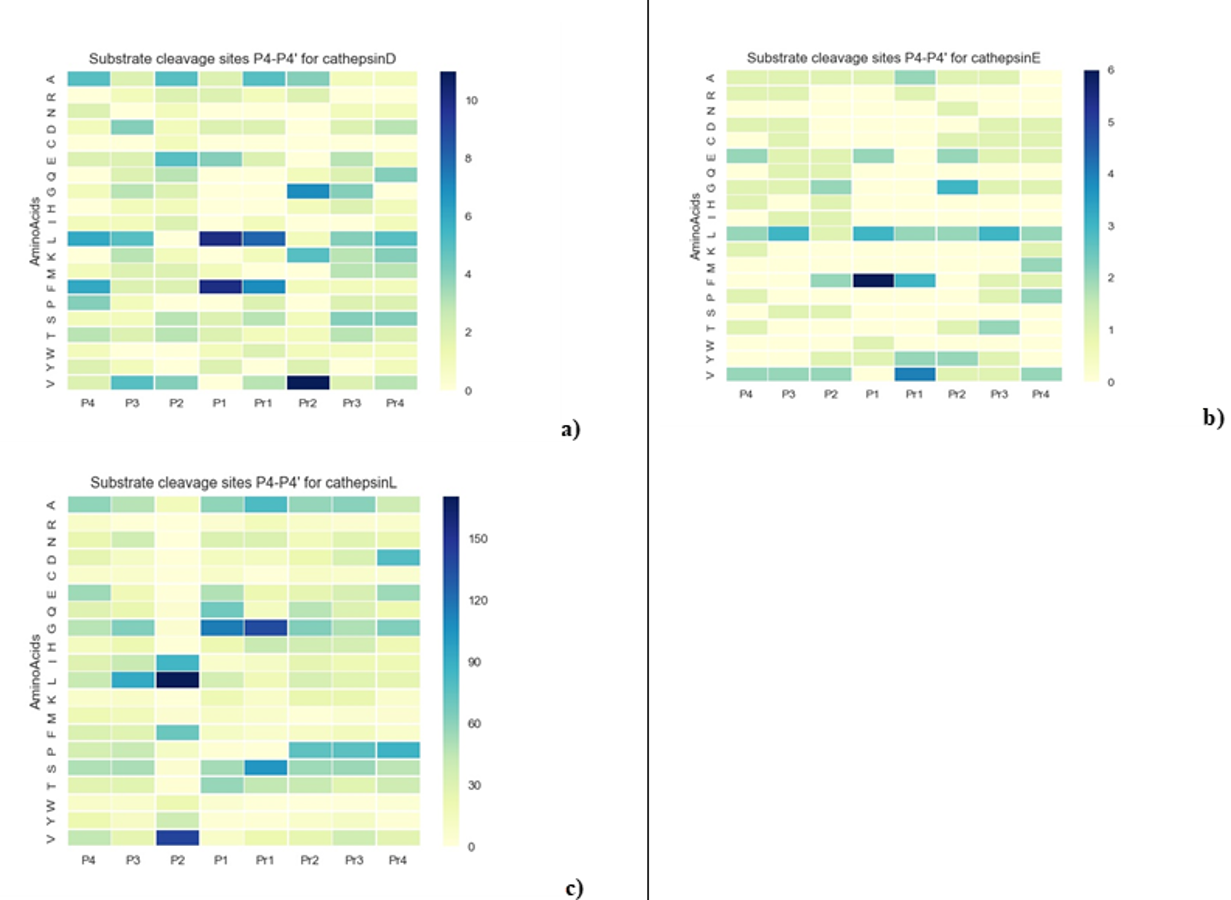

Supplement: S1 Fig — Substrate cleavage sites for P4-P4’ local window for cathepsins: a) cathepsin D; b) cathepsin E; c) cathepsin L. The cumulative amino acid occurrences in P4–P4’ were calculated and displayed in the form of a two-dimensional heat map. The scissile peptide bond was between sites P1 and P1’. (TIF) [file pone.0199270.s011.tif]

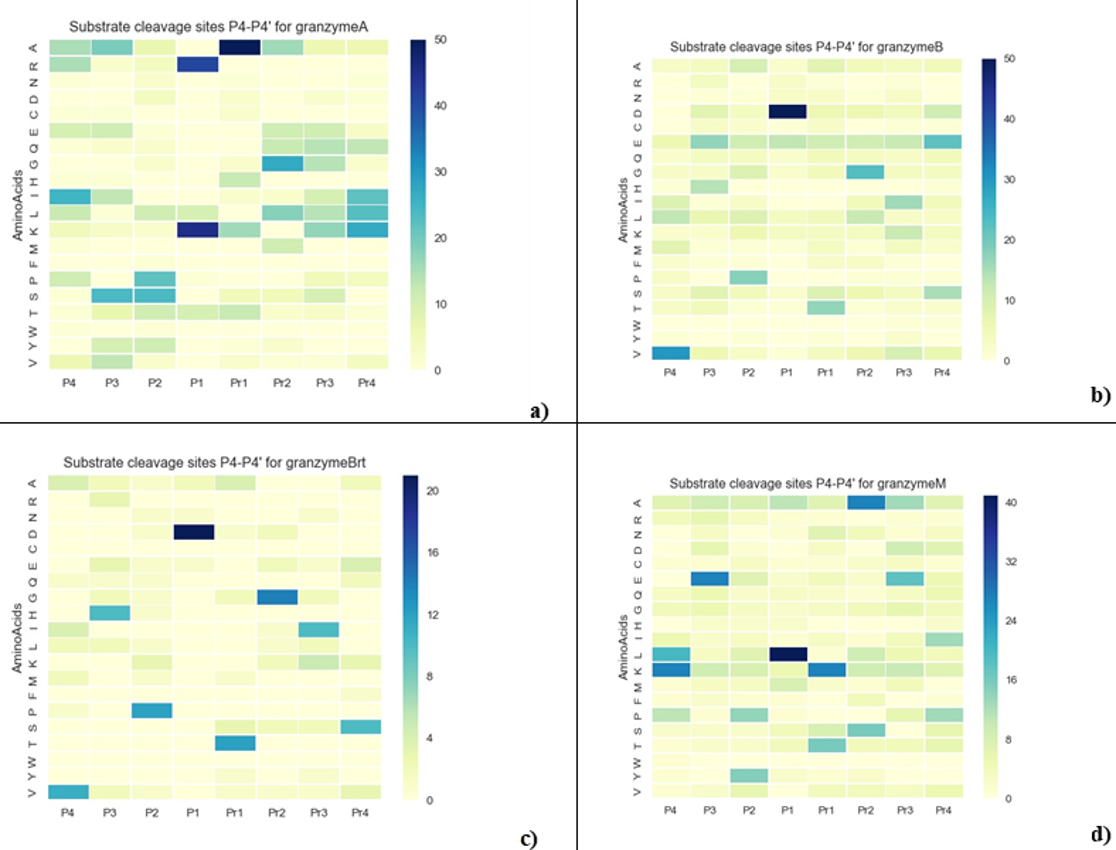

Supplement: S2 Fig — a) granzyme A; b) granzyme B; c) granzyme B (rodent-type); d) granzyme M. The cumulative amino acid occurrences in P4–P4’ were calculated and displayed in the form of a two-dimensional heat map. The scissile peptide bond was between sites P1 and P1’. (TIF) [file pone.0199270.s012.tif]

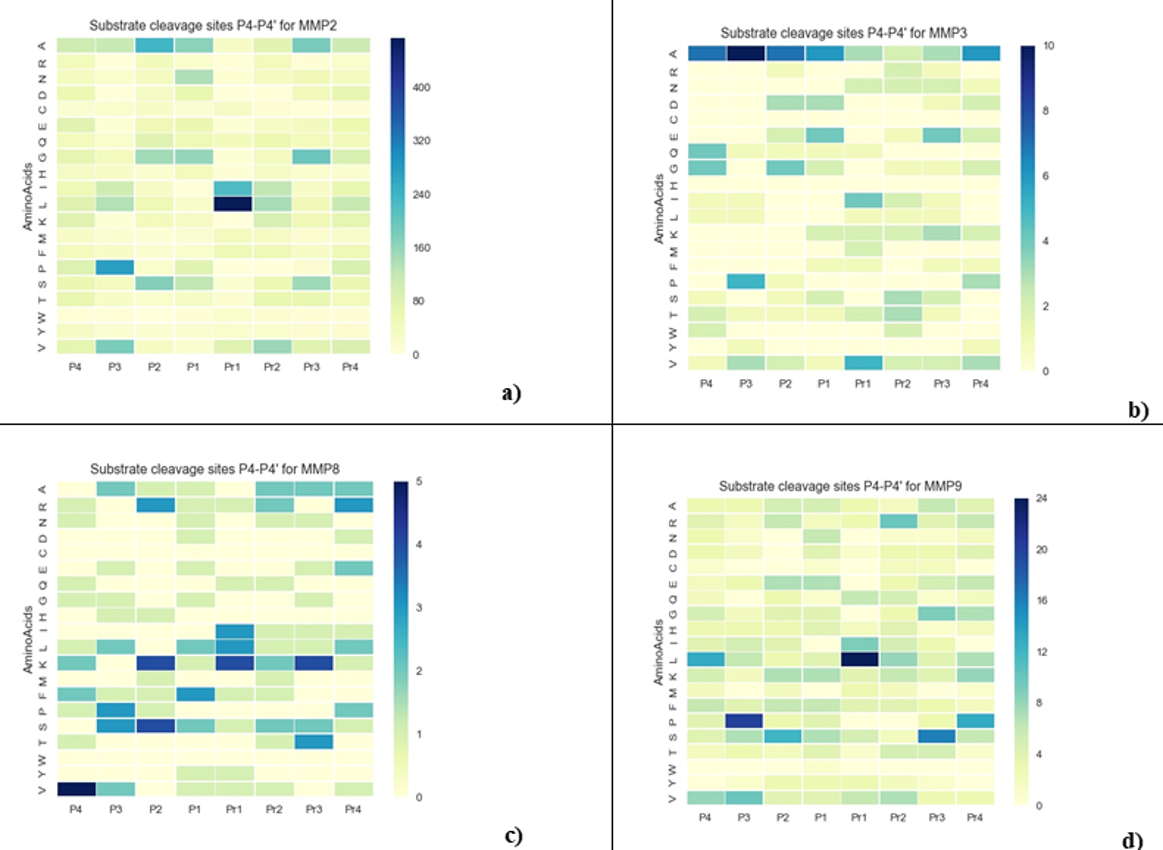

Supplement: S3 Fig — a) MMP2; b) MMP3; c) MMP8; d) MMP9. The cumulative amino acid occurrences in P4–P4’ were calculated and displayed in the form of a two-dimensional heat map. The scissile peptide bond was between sites P1 and P1’. (TIF) [file pone.0199270.s013.tif]
